# Gonococcal Genetic Island in the Global *Neisseria gonorrhoeae* Population: A Model of Genetic Diversity and Association with Resistance to Antimicrobials

**DOI:** 10.3390/microorganisms11061547

**Published:** 2023-06-10

**Authors:** Dmitry Kravtsov, Dmitry Gryadunov, Boris Shaskolskiy

**Affiliations:** Center for Precision Genome Editing and Genetic Technologies for Biomedicine, Engelhardt Institute of Molecular Biology, Russian Academy of Sciences, 119991 Moscow, Russia; solo13.37@yandex.ru (D.K.); grad@biochip.ru (D.G.)

**Keywords:** *Neisseria gonorrhoeae*, gonococcal genetic island, genetic diversity, antimicrobial resistance

## Abstract

The aim of this work was to study the genetic diversity of the gonococcal genetic island (GGI) responsible for the type IV secretion system (T4SS) and the association of a functionally active GGI with antimicrobial resistance. An analysis of the GGI in a sample of 14,763 genomes of *N. gonorrhoeae* isolates from the Pathogenwatch database collected in 1996–2019 from 68 countries was performed. A model of GGI’s genetic diversity that divides the global gonococcal population into fifty-one GGI clusters and three GGI superclusters based on the allele type of the *traG* gene and substitutions of the *atlA* and *ych* genes for *eppA* and *ych1* has been proposed, reflecting differences among isolates in the T4SS functionality. The NG-MAST and MLST typing schemes (with accuracies of 91% and 83%, respectively) allowed the determination of both the presence of a GGI and the GGI cluster and, correspondingly, the structure of the GGI and the ability to secrete DNA. A statistically significant difference in the proportion of *N. gonorrhoeae* isolates resistant to ciprofloxacin, cefixime, tetracycline, and penicillin was found when comparing populations with a functional and a non-functional GGI. The presence of a functional GGI did not affect the proportion of azithromycin-resistant isolates.

## 1. Introduction

Gonorrhea is one of the most common sexually transmitted infections in the world; its causative agent is the Gram-negative microorganism *N. gonorrhoeae*. According to the WHO, about 87 million new cases of this disease are registered in the world every year [1]. The public health importance of gonorrhea is defined by several aspects, such as its continued high morbidity rate, predominant distribution among reproductive-age individuals and its pronounced negative impact on fertility, increased risk of co-infection with other sexual transmitted infections, absence of a gonococcal vaccine, and the progressive resistance of *N. gonorrhoeae* to antimicrobials [2].

The history of the use of antibiotics for *N. gonorrhoeae* therapy shows the sequential emergence of resistance to all the drugs used, which can be explained by the exceptional ability of *N. gonorrhoeae* to change its genetic material due to different mechanisms, including horizontal gene transfer (HGT) [3]. A high frequency and efficiency of transformation, which are most likely the key mechanisms of chromosomal DNA transfer for *N. gonorrhoeae* [4], are due to the natural competence of these bacteria [5,6]. To release DNA into the external environment, the gonococcus uses a type IV secretion system (T4SS) encoded by the genes of the gonococcal genetic island (GGI). In general, T4SSs are widespread in both Gram-positive and Gram-negative microorganisms and are subdivided into three families according to their biological functions [7]: (1) DNA conjugation, (2) protein transfer to a recipient cell (direct contact with the recipient cell is required), and (3) DNA secretion into the external environment (no contact with the cell is required). The gonococcal T4SS belongs to the latter family and releases single-stranded DNA into the medium in a contact-independent manner [8]. The DNA is then specifically recognized by the recipient pili by the DNA uptake sequences (DUS) and is recombined into the genome. In addition, the GGI plays an important role in biofilm formation due to the large contribution of “sticky” DNA in the construction of the biofilm matrix [9]. In a recent study by Youngblom et al. [10], it was found that the presence/absence of GGI is linked to the phylogeny of the *N. gonorrhoeae*, indicating that isolates with and without this element form genetically differentiated groups and may inhabit different niches in the human body. Moreover, these researchers suggested that isolates without GGI are less likely to acquire new gene variants via HGT.

The GGI is ~59,000 nucleotides long and includes 66 genes [6,11,12]. Like all genetic islands, the GGI is a mobile element, and it itself was obtained by a horizontal transfer (as evidenced by the reduced GC content of 43% as compared to the 52% average value for gonococcus and by a much lower number of DUS per unit length [13]). The XerCD site-specific recombination system is responsible for GGI mobility, cutting/inserting the island at the *difA* and *difB* flanking sites [14]. To identify the genes required for GGI functioning, targeted mutagenesis of the GGI loci was performed, followed by measurements of single-stranded DNA fluorescence in the supernatant [5,15,16]. As a result of these works, it was found that only 21 (*traG*, *atlA*, *traI*, *parA*, *parB*, *traK*, *traV*, *traB*, *traD*, *ltgX*, *yag*, *traL*, *traE*, *dsbC*, *traC*, *traW*, *traU*, *trbC*, *traN*, *traF*, and *traH*) of 66 island genes are required to preserve the GGI DNA-secreting ability. In this paper, an island encoding T4SS that is potentially capable of secreting DNA (i.e., without damage in these 21 genes) is referred to as a functional island. To date, the role of all the proteins encoded by the GGI genes in DNA secretion has not been established. Nevertheless, it is known that the *traK*, *traV*, and *traB* genes encode proteins of the core complex, and the products of the *traI*, *parA*, and *parB* genes refer to the DNA-processing proteins. The *traG* gene encodes a membrane protein with five transmembrane domains that can interact with other T4SS proteins [11]. The TraG protein was found to affect TraH protein localization, possibly by means of stabilizing or facilitating TraH transport to the gonococcal outer membrane [17].

Significant variations in the GGI in the *traG–atlA–ych–exp1–cspA–exp2* gene region have been reported in the literature. Three different classes of GGI depending on the *traG* variant and the presence of the *atlA* gene have been identified, and it has been shown that they differ in the level of DNA secretion into the extracellular medium [18].

In the work of Harrison et al. [19], in a sample of 288 isolates (most of which were from the USA), an association was found between the presence of a GGI and an increase in the proportion of isolates with resistance to cefixime, penicillin, tetracycline, and ciprofloxacin and a decrease in the proportion of isolates resistant to azithromycin. In our previous work in a sample of 150 *N. gonorrhoeae* genomes belonging to the *Neisseria gonorrhoeae* multiantigen sequence typing (NG-MAST) types that are predominant worldwide (225, 1407, 2400, 2992, and 4186) and the most common Russian type, 807, a relationship between isolate susceptibility to ciprofloxacin, penicillin, and tetracycline and the presence of lesions in the GGI genes necessary for DNA secretion was established [20]. Given the importance of analyzing antimicrobial resistance, these results should be validated using global datasets of *N. gonorrheae* genomes.

In the present work, we performed a versatile analysis of the gonococcal genetic island using 14,763 genomes of *N. gonorrhoeae* isolates from all over the world. The tasks of this work were to study the genetic diversity of the GGI, to determine the heterogeneity of the GGI in isolates with the same molecular types from traditional NG-MAST and Multi-locus sequence typing (MLST) schemes and to establish the relationship between the presence of a functional GGI and resistance to antimicrobial drugs.

## 2. Materials and Methods

### 2.1. Genomes from the Databases

All *N. gonorrhoeae* genomes available in the Pathogenwatch database at the time of download (https://pathogen.watch/collections/all?organismId=485, accessed on 15 November 2022) were taken to obtain the genomic data and the corresponding metadata. The *N. gonorrhoeae* genomes belonged to various collections from around the world (isolates prior to 1996 were not counted). A total of 29 collections were taken, including genomes of 14,763 isolates from 68 countries (Supplementary Figure S1, Supplementary Table S1). The final sample also comprised 61 genomes of the Russian isolates described earlier [21,22].

The Supplementary Table S1 included the following metadata for each isolate: displayname according to Pathogenwatch database record; isolation year; country of origin; strain; collection label; EMBL/Genbank Bioproject No.; EMBL/Genbank Accession No.; EMBL/Genbank Biosample No.; results of antimicrobial susceptibility testing for spectinomycin, tetracycline, azithromycin, cefixime, ceftriaxone, ciprofloxacin, and penicillin; molecular typing data (NG-MAST, MLST, NG-STAR), antimicrobial resistance determinants (mutations in the 16S rRNA/23S rRNA genes, presence of *blaTEM* and/or *tetM* plasmids, substitutions in the ribosomal proteins (*rpsJ*, *rpID* genes), porin influx pump (*porB* gene), MtrCDE efflux pump (*mtrR*, *mtrC*, *mtrD* genes), penicillin-binding proteins (*penA*, *ponA* genes), DNA-gyrase, and IV type topoisomerase (*gyrA*, *parC* genes)); GGI characteristics according to the results of pubMLST Typing, phylogenetic analysis (as described in Section 2.4), and clustering (as described in Section 2.5); and testing for GGI functionality (as described in Section 2.6).

### 2.2. Genome-Based Prediction of Resistance to Antimicrobial Drugs

The data on resistance to azithromycin, cefixime, ceftriaxone, ciprofloxacin, penicillin, and tetracycline for *N. gonorrhoeae* isolates were obtained from the Pathogenwatch database (Supplementary Table S1). The minimum inhibitory concentration (MIC) values were calculated by an algorithm described earlier [23]. Values for resistant (R), intermediate (I), and susceptible (S) isolates follow the EUCAST clinical breakpoints.

### 2.3. Search for GGI Alleles in Genomes—Multiple GGI Alignment

NCBI BLAST+ tools, v.2.13.0 (https://anaconda.org/bioconda/blast, accessed on 14 January 2023), were used to search for GGI alleles across all contigs. The MLST software v.2.23 (https://github.com/tseemann/mlst, accessed on 15 January 2023) was used for the typing of GGI alleles. All GGI gene alleles, both known and new, were concatenated to obtain multiple alignments.

### 2.4. Construction of Phylogenetic Trees

The removal of ambiguously aligned regions was performed with bmge v.1.12 [24] using the default settings. The same was performed for multiple alignments obtained from the concatenated loci included in the typing schemes according to NG-MAST (sequencing of the *porB* and *tbpB* genes [25]) or MLST (sequencing of the *abcZ*, *adk*, *aroE*, *fumC*, *gdh*, *pdhC*, and *pgm* genes [26]) protocols. Gene sequence alignments were performed in AliView [27]. Then, a maximum-likelihood phylogenetic tree was constructed using IQ-TREE v.1.6.12 [28] with ultrafast bootstrap options [29] and 1000 iterations in 3 runs. The tree for the concatenated GGI-composing genes was visualized using GrapeTree v.1.5.0 [30], and the trees for the concatenated loci included in the NG-MAST and MLST typing schemes were visualized using Dendroscope v.3.8.5 [31].

### 2.5. Clustering of GGI Sequences from the Multiple Alignment

To cluster genetic islands, the square distance matrix (*p*-distance) between sequences from the multiple GGI alignment obtained in Section 2.3 was first calculated. Then, the clusters were found using R package dbscan v.1.1-11 [32], and the mean value over the entire distance matrix (*p*-distance = 0.0106) was set as a limiting parameter. A cluster was assigned a number if the number of genomes in it was at least 10.

### 2.6. Searching for Mutations Resulting in a Loss of GGI Functionality

The analyzed genomes were mapped to the MS11 reference genome (Genbank assembly accession number GCF_019212165.1), which possesses a functional GGI, and each GGI gene was translated and screened for the presence of frameshifts and premature stop codons. Variant-calling was performed for the detected lesions using vSNP v.3.11 (https://github.com/USDA-VS/vSNP3, accessed on 11 February 2023). Raw reads of the corresponding genomes from the ENA database (https://www.ebi.ac.uk/ena/browser/home, accessed on 11 February 2023) were mapped to the same MS11 genome. Thus, all the lesions were validated by taking into account the depth of reads, and the invalid ones were discarded.

### 2.7. Nucleotide Diversity θπ and Tajima’s D Test

The nucleotide diversity θπ for the GGI genes was calculated using the Tamura–Nei 1993 model [33] using the tn93 v.1.0.11 bash utility (https://github.com/veg/tn93, accessed on 25 February 2023). θπ is a characteristic that shows the average number of nucleotides that differ in two randomly selected sequences in a multiple alignment. The value of Tajima’s D test [34] was calculated using the tajimas_d v.2.0.0 utility (https://github.com/not-a-feature/tajimas_d, accessed on 25 February 2023). Negative values of Tajima’s D indicate that the population is expanding or that the population relatively recently passed through the bottleneck. Positive values of Tajima’s D indicate subdivision or splitting of the population. Tajima’s D values close to zero indicate a stable population size.

### 2.8. dN/dS Calculation

To calculate the ratio of nonsynonymous amino acid substitutions to synonymous ones (dN/dS), the snpgenie v.1.0 (https://github.com/chasewnelson/SNPGenie/blob/master/snpgenie_within_group.pl, accessed on 2 March 2023) program was used. The dN/dS ratio exceeds unity if natural selection promotes changes in the protein sequence, whereas a ratio less than 1.0 is expected if natural selection suppresses changes in the protein [35,36].

### 2.9. Statistical Processing of the Data

The significance of differences in the frequency of drug-resistant isolates was determined with a χ2 test (Pearson’s chi-squared test for count data) using R package stats v.4.2.2 (https://www.r-project.org/, accessed on 10 March 2023).

## 3. Results

### 3.1. GGI Genetic Diversity

An analysis of 14,763 genomes revealed that 9776 (66.2%) genomes had a GGI, 34 (0.2%) had a truncated GGI, and a GGI was absent in 4953 (33.6%) genomes (Table S1).

**Figure 1:**
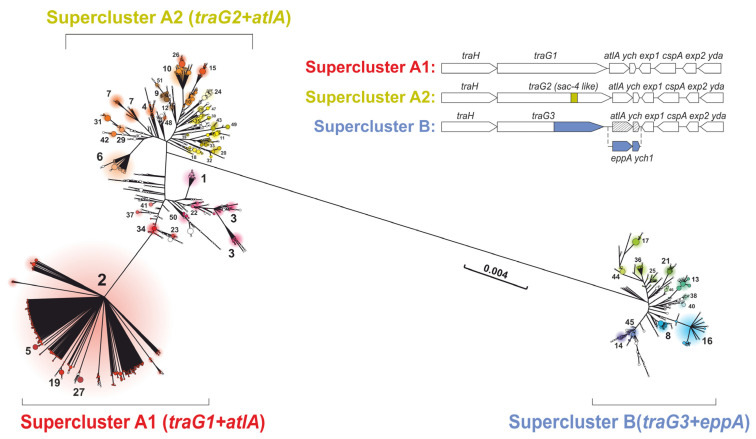
Phylogenetic tree constructed for the GGI genes of 9776 genomes. Three superclusters, (A1), (A2), and (B), and fifty-one clusters are shown. Clusters are marked by colors. On the upper right, the characteristic differences in the GGI genes (mainly in the *traG*, *atlA/eppA*, and *ych/ych1* genes) between the superclusters are shown.

A maximum-likelihood phylogenetic tree was constructed for the genomes with a GGI (Figure 1). The phylogenetic analysis revealed that the analyzed genomes can be divided into three phylogenetic superclusters, (A1), (A2), and (B), which were primarily associated with different *traG* isoforms and downstream genes from *traG* to *exp2*. The isolates of superclusters (A1) and (A2) possessed the *atlA* and *ych* genes, while the *eppA* and *ych1* genes were absent. In contrast, supercluster (B) included isolates with the *eppA* and *ych1* genes instead of *atlA* and *ych*. In addition to these differences detected in 99–100% of isolates, supercluster (A2) had a second repeat of the *yecB* gene in 75% of the isolates under study, and the *cspA* gene was found in only 60% of the isolates.

**Figure 2:**
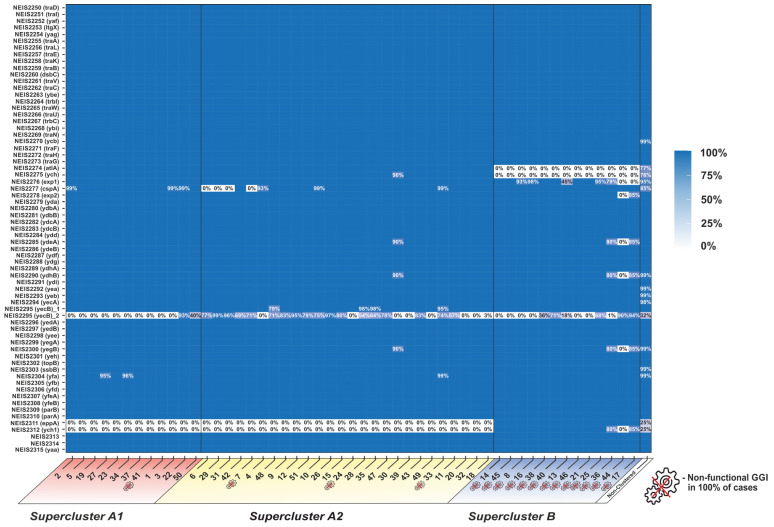
Heatmap of the presence of GGI genes in different clusters. Clusters are listed according to their entry into the superclusters. The “crossed gear” symbol indicates clusters in which 100% of the isolates are unable to secrete DNA due to changes in the GGI structure. The color intensity and numerical value (%) show the proportion of a particular GGI gene present in each of the clusters.

The clustering of GGI into 51 clusters (shown in Figure 1) had several goals. First, it allowed us to reduce the sampling size under study: it is more convenient to work with 51 clusters than with more than 9500 genomes. Second, we were able to reduce the sampling imbalance: it was possible to obtain characteristics for each cluster and evaluate them despite the fact that there could be more isolates in one cluster than in another. The third reason for clustering was the possibility of isolating sub-populations with similar GGI structure. In particular, the isolates of clusters 6, 7, 29, and 31, which belonged to supercluster (A2), were characterized by the absence of the *cspA* gene, which, however, did not lead to the loss of GGI functionality in these isolates. One hundred percent of the genomes in supercluster (B) were characterized by the presence of a non-functional gonococcal island. The proportion of non-clustered isolates was 5.8% of all isolates with a full-sized GGI (Figure 2, Supplementary Table S2).

**Figure 3:**
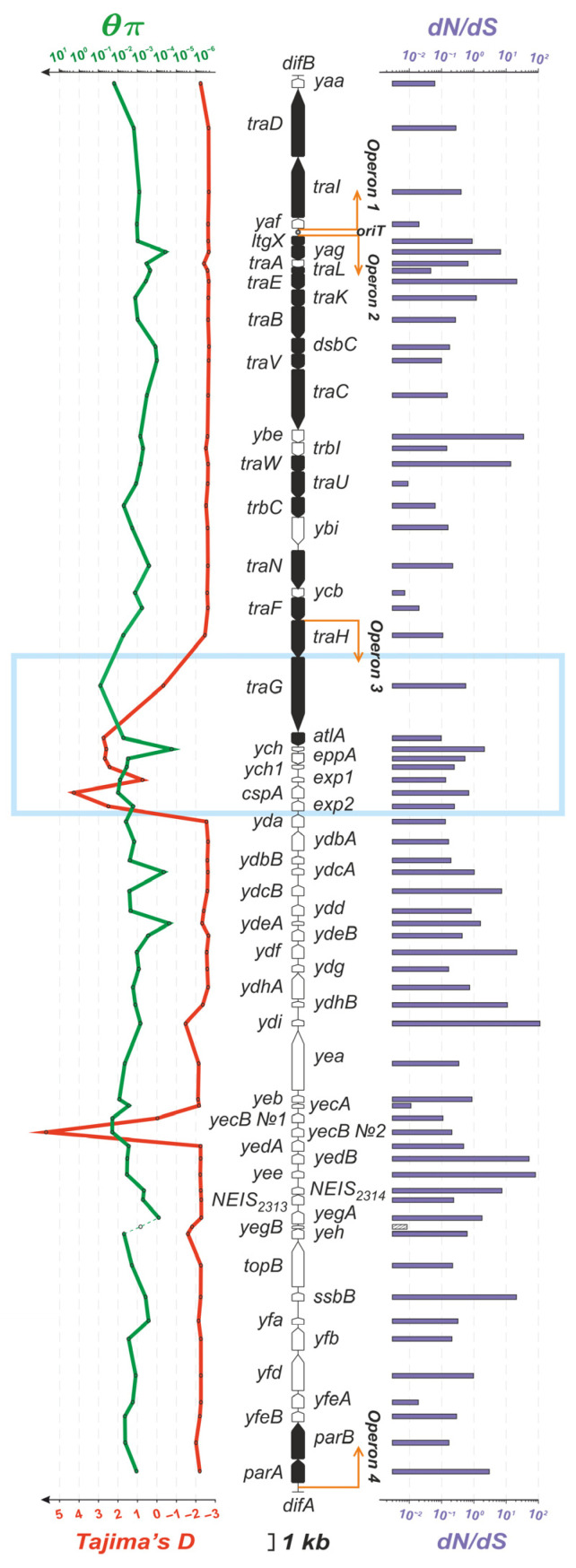
Characteristics of GGI genes in *N. gonorrhoeae* genomes: the values of θπ (green line on the left), Tajima’s D statistic test (red line on the left), and dN/dS (purple bars on the right). The genes required to preserve the GGI DNA-secreting ability are colored in black. The blue rectangle marks the most polymorphic region within the GGI.

The nucleotide diversity values θπ (Figure 3, green line) and Tajima’s D statistic test values (Figure 3, red line) were calculated for the GGI genes in the analyzed sample. Tajima’s D value for the entire GGI was −2.066, which suggests the expansion of the *N. gonorrhoeae* population with the GGI. When calculating separate Tajima’s D values for superclusters, we obtained the values of −2.622 for (A1), −0.945 for (A2), and −1.253 for (B) (Supplementary Table S3). Thus, meaningful direct selection may be noted only for supercluster (A1). For the *atlA*, *ych*, *cspA*, *exp2*, *yecB*, *eppA*, and *ych1* genes, the calculated value of Tajima’s D was greater than 2.4, indicating that the global *N. gonorrhoeae* population was separated namely at these most polymorphic GGI loci (Figure 3, blue rectangle).

The *traG* gene appeared to be the most polymorphic (θπ = 0.0783), which can be explained by the existence of three isoforms, i.e., *traG1* in supercluster (A1), *traG2* in (A2), and *traG3* in (B) (see Figure 1 and the TraG protein alignment in Supplementary Figure S2). The θπ values for *traG* were significantly lower within each of the superclusters (0.00154, 0.00557, and 0.00401), indicating that the *traG1*, *traG2*, and *traG3* isoforms are conservative. In addition, this conservatism inside the superclusters was confirmed by a dN/dS value close to 1. The *yaa* gene was also polymorphic (θπ = 0.0155) with dN/dS = 0.06 (stabilizing selection was observed; i.e., changes were not evolutionarily beneficial and isolates with mutations were not fixed in the population). Among the genes required to preserve the functional activity of the GGI, the first ten most variable genes included *traH*, *atlA*, and *trbC* with θπ = 0.0052, 0.0051, and 0.0050, respectively (Supplementary Table S3).

Among the 21 genes required for functional GGI activity, the highest dN/dS values were observed for the periplasmic protein gene *yag* (dN/dS = 6.9) and the chromosome-partitioning protein gene *parA* (dN/dS = 3.1). The essential GGI genes did not stand out as a separate group, either by nucleotide diversity or by the ratio of nonsynonymous to synonymous substitutions, demonstrating a large scattering in both the θπ and dN/dS values (Supplementary Table S3).

### 3.2. Relationship between the GGI Phylogeny and NG-MAST and MLST Types

**Figure 4:**
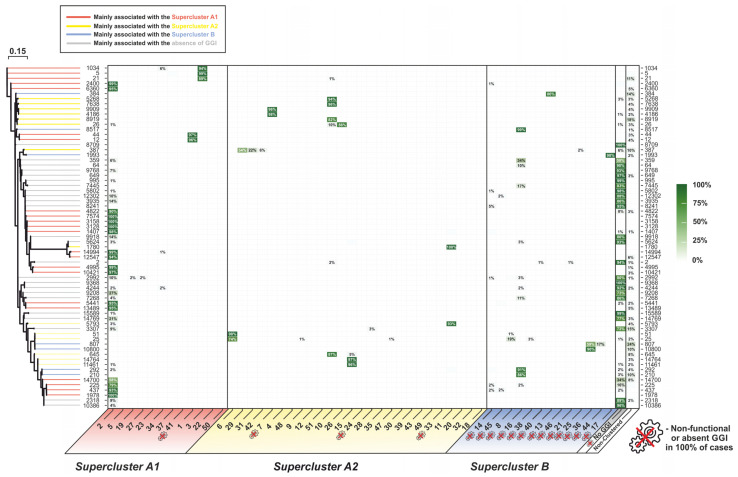
Distribution of NG-MAST types by GGI clusters and heatmap of the occurrence of isolates within NG-MAST types. The phylogram for the most frequent NG-MAST types is shown on the left. The “crossed gear” symbol indicates GGI clusters in which 100% of the isolates were unable to secrete DNA due to changes in the GGI structure and isolates without a GGI. The color intensity and numerical value (%) show the proportion of a GGI cluster within an NG-MAST type.

**Figure 5:**
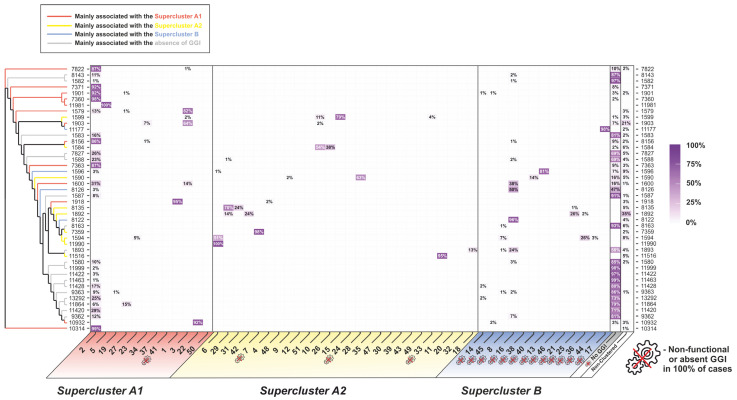
Distribution of MLST types by GGI clusters and heatmap of the occurrence of isolates within MLST types. The phylogram for the most frequent MLST types is shown on the left. The “crossed gear” symbol indicates GGI clusters in which 100% of the isolates were unable to secrete DNA due to changes in the GGI structure and isolates without a GGI. The color intensity and numerical value (%) show the proportion of a GGI cluster within an MLST type.

To consider the relationships between GGI phylogeny and known molecular typing schemes, we calculated the proportions of GGI clusters within the most abundant NG-MAST and MLST types (Figure 4 and Figure 5, respectively, and Supplementary Table S4).

The results obtained showed that, for 91% of isolates, the NG-MAST type indicated the presence or absence of a GGI and the GGI cluster if the GGI was present. Thus, more than 97% of the isolates of the widespread NG-MAST 1407 belonged to cluster 2 of supercluster (A1), and more than 97% of the isolates of NG-MAST 4186 belonged to cluster 7 of supercluster (A2); 100% of the isolates of NG-MAST types 8709 and 9368 had no GGI. The observed heterogeneity in the distribution of GGI clusters by NG-MAST types usually existed only within one supercluster. However, there were exceptions in 8%, such as NG-MAST type 25, for which 73% of the isolates belonged to cluster 6 of supercluster (A2) and 18% belonged to cluster 8 of supercluster (B) (Figure 4).

A similar pattern was observed for MLST types, although it was less pronounced due to the high conservativity of the loci used for the typing. In 83% of cases, a GGI cluster could be identified by the MLST type. For example, for genomes of the world’s most common MLST type, 1901, 92.2% of the genomes belonged to cluster 2 of supercluster (A1); 0.9% belonged to cluster 14 of supercluster (B), and 3.3% had no GGI. In contrast, for the isolates from MLST type 9363, 8.8% of the genomes belonged to cluster 2 of supercluster (A1); 1.9% belonged to cluster 16 of supercluster (B), and 85.8% had no GGI (Figure 5).

It should be noted that, in the phylogenetic trees for both NG-MAST (Figure 4) and MLST (Figure 5), the branches with different GGI superclusters were generally not arranged according to the NG-MAST or MLST phylogeny clades (shown on the left side of the figures). In the NG-MAST or MLST trees, multiple alternations of branches and clades belonging to different superclusters can be seen when traversing the tree branches.

In addition, the heterogeneity characteristics of GGI clusters by NG-MAST and MLST types were calculated. For this purpose, we assembled all NG-MAST or MLST types for the isolates of the selected GGI cluster into a single alignment and calculated the average number of nucleotides that differed from the dominant NG-MAST or MLST type in the cluster per nucleotide sequence of concatenated NG-MAST or MLST loci (Supplementary Table S5, Supplementary Figures S3 and S4). For all three superclusters, a higher heterogeneity in NG-MAST types, on average 113 nucleotides/sequence, was observed within large clusters (200 isolates or more). For example, in cluster 2, the heterogeneity was 215 nucleotides/sequence; within this cluster, 12% of the isolates belonged to NG-MAST 1407; 9% belonged to NG-MAST 5441, and 6% belonged to NG-MAST 2400. For MLST types, due to the greater conservativity of the genes used, the calculated heterogeneity rates were lower (0.8 nucleotides/sequence), but the form of heterogeneity distribution across the clusters was similar (Supplementary Figures S3 and S4).

Thus, in most cases, it was possible to determine the GGI cluster by NG-MAST type, while phylogenetically distant NG-MAST sequence types may present within the same GGI cluster (knowing the NG-MAST type, one can predict the GGI cluster and approximate GGI alleles, but the reverse is impossible). Obtaining information on the GGI cluster number allows one to predict both the DNA secretion ability and the allelic composition of the GGI genes, including *traG*.

### 3.3. Association between GGI Type and Antimicrobial Resistance

We performed a pairwise comparison of antimicrobial susceptibility distributions between isolates with a functional GGI and isolates without a GGI and also between isolates with a functioning and non-functioning GGI. Background data for the comparisons are given in Supplementary Table S6.

**Figure 6:**
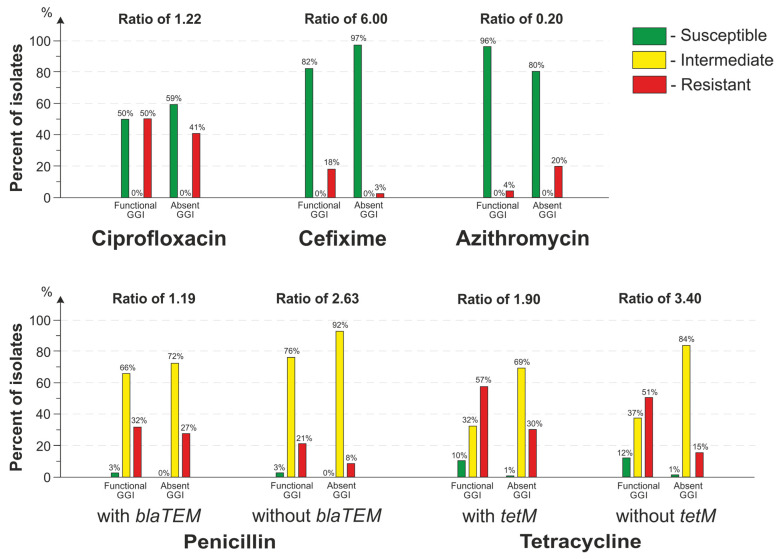
Distribution of isolates by antimicrobial susceptibility depending on the presence of a functional GGI or absence of a GGI in the genome. Ratio was calculated as the proportion of resistant isolates with a functional GGI to the proportion of resistant isolates without a GGI.

It was shown that there was a statistically significant (*p* << 0.001) association between the presence of a functional GGI and a decreased susceptibility to penicillin, tetracycline, cefixime, and ciprofloxacin and an increased susceptibility to azithromycin. In the presence of a functionally active GGI, the proportion of cefixime-resistant isolates was 6.00-fold greater as compared with the isolates without a GGI; the proportion of ciprofloxacin-resistant isolates was 1.22-fold greater (Figure 6).

It is known that the resistance to penicillin and tetracycline is affected by both chromosomal determinants and *blaTEM* and *tetM* plasmid determinants. At the same time, it has been experimentally proven that GGI-mediated DNA transfer occurs by DNA secretion and natural transformation rather than by conjugation [8]. Therefore, to account for the GGI contribution to horizontal transfer of resistance determinants, we compared the proportions of resistant isolates both with and without *blaTEM* and *tetM* plasmids.

When the isolates with and without *blaTEM* plasmids were considered together, the proportion of penicillin-resistant isolates was 1.19-fold larger as compared with isolates without a GGI. When only isolates without plasmids were considered, the proportion of penicillin-resistant isolates was 2.63-fold larger. Similarly, when isolates with and without *tetM* plasmids were considered together, the proportion of tetracycline-resistant isolates was 1.90 times higher as compared with isolates without a GGI. When only isolates without *tetM* plasmids were considered, the proportion of tetracycline-resistant isolates was 3.4 times higher (Figure 6).

An interesting observation was that the azithromycin resistance fell out of the identified trend; that is, the proportion of resistant isolates with a functional GGI was 5.00 times lower than that of the isolates without a GGI (Figure 6).

**Figure 7:**
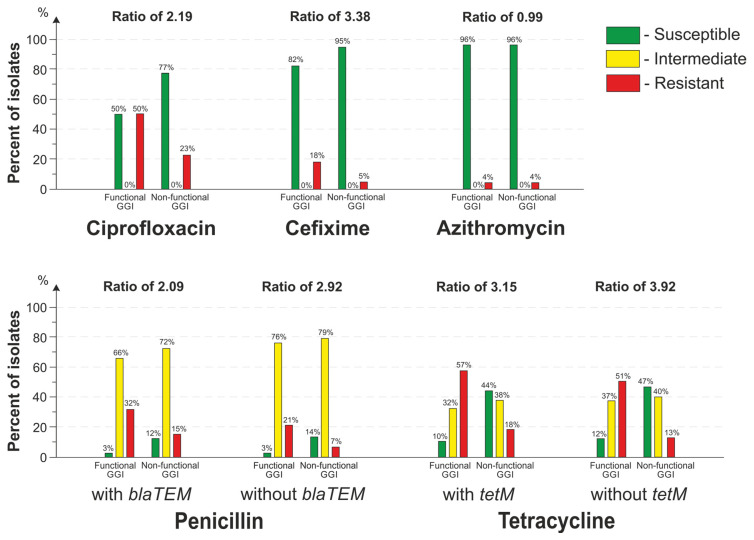
Distribution of isolates by antimicrobial susceptibility depending on the ability of the GGI to secrete DNA. Ratio was calculated as the proportion of resistant isolates with a functional GGI to the proportion of resistant isolates with a non-functional GGI.

The results of the comparison of antimicrobial susceptibility distributions for the isolates with a functional and a non-functional GGI are shown in Figure 7. The analysis revealed a statistically significant (*p* << 0.001) relationship between the presence of a functional GGI and resistance to penicillin, tetracycline, cefixime, and ciprofloxacin.

In the presence of a functional GGI, the proportion of cefixime-resistant isolates was 3.38-fold higher than that of the isolates with a non-functional GGI; the proportion of ciprofloxacin-resistant isolates was 2.19-fold higher (Figure 7).

When isolates with and without *blaTEM* plasmids were considered together, then the proportion of penicillin-resistant isolates was 2.09 times higher as compared with the isolates with a non-functional GGI. When only isolates without *blaTEM* plasmids were considered, the proportion of penicillin-resistant isolates was 2.92 times higher. Likewise, when isolates with and without *tetM* plasmids were considered together, the proportion of tetracycline-resistant isolates was 3.15 times higher as compared with isolates with a non-functional GGI. When only isolates without *tetM* were considered, the proportion of tetracycline-resistant isolates was 3.92 times higher.

The proportion of isolates resistant to azithromycin did not change when comparing isolates with a functional and a non-functional GGI.

## 4. Discussion

According to the literature data, a GGI was found in about 80% of *N. gonorrhoeae* isolates and in 17% of *N. meningitidis* isolates and was not found in commensal *Neisseria* species [13]. In this work on a sample of 14,763 *N. gonorrhoeae* isolates from 68 countries, we showed that 66% of the *N. gonorrhoeae* isolates possessed a GGI, with 22% of them having alterations and breakages resulting in the loss of the ability to secrete DNA.

Previously, Dillard and Seifert [18] used a sample of 115 isolates, including isolates with and without a GGI, to distinguish three different classes of GGI based on *traG* gene polymorphism: class I—*traG1* + *atlA*, class II—*traG2(sac-4)* + *atlA*, and class III—*traG3* without *atlA*, which differed in the level of DNA secretion into the extracellular medium. Later, Kohler et al. [8] found that isolates possessing a class III GGI had the *eppA* gene in place of *atlA* and the *ych1* gene instead of *ych* and also lacked the *exp1* gene; the inability of a GGI carrying the *traG3* + *eppA* genes to secrete DNA was confirmed. Callaghan et al. proposed that peptidoglycanase AtlA is necessary for secretion because it creates an opening in the cell wall to provide assembly of the whole system on the membrane; at the same time, endopeptidase EppA is not able to replace peptidoglycanase AtlA [11]. It seemed important to us to trace these features of GGI in the global population of *N. gonorrhoeae.*

In this work, based on a phylogenetic analysis and clustering, we proposed a model describing the division of the global *N. gonorrhoeae* population with a GGI into three superclusters, (A1), (A2), and (B), consisting of a total of 51 clusters. Thus, the previously described division into three GGI classes [8,18] was confirmed using 14,763 *N. gonorrhoeae* genomes from around the world. Information on the attribution to a cluster allowed us to make a conclusion about the GGI activity, GGI gene composition, *traG* gene type, and allelic composition of other genes. Moreover, within superclusters (A1) and (A2), the majority of isolates possessed a functional GGI with the *atlA* and *ych* genes, while all the cluster isolates included in supercluster (B) had a non-functional GGI with the *eppA* and *ych1* genes.

Based on Tajima’s D values, the *N. gonorrhoeae* population belonging to supercluster (A1) (NG-MAST 1407, 5441, 2400, 1034, 21, 5, etc.) has an advantage in natural selection. The GGI genes, whose lesions/critical changes result in the loss of the ability to secrete DNA, did not stand out as a separate group, either in nucleotide diversity or in the dN/dS values (Figure 3). This can probably be explained by differences in the biological functions of these genes.

In 91% of cases, it was possible to determine the type of GGI by the NG-MAST type. At the same time, phylogenetically distant NG-MAST sequence types can be found within the same GGI genetic cluster. Thus, by knowing the NG-MAST type, one can predict the GGI cluster and approximate GGI alleles, but the reverse is impossible. The obtained results confirmed the correctness of the assumption of our previous work carried out using 25 genomes for each of the most frequent NG-MAST types (225, 807, 1407, 2400, 2992, and 4186) about the relationship between the NG-MAST type and the GGI genetic characteristics [20]. Determination of the NG-MAST type and prediction of GGI functionality can be used to assess the ability of a *N. gonorrhoeae* isolate to form biofilms. Biofilms are known to confer increased drug resistance levels and are one of the causes of chronic infections – under these conditions, bacteria can survive longer after antibiotic exposure. A significant difference in the ability to form biofilms produced by the MS11 strain with functional GGI and the mutant MS11 possessing non-functional GGI (with a deletion of the essential *traB* gene) was demonstrated [9]. It was found that the ability to secrete single-stranded DNA plays an essential role at the initial stages of biofilm formation; it is assumed that T4SS determines the colonization and transmission rates of a given strain. Thus, knowledge of the GGI functionality may have important clinical relevance in the future.

It should be noted that clades and branches on the phylogenetic tree, both for NG-MAST and MLST, belonging to different superclusters alternated with one another. This means that phylogenetic closeness by NG-MAST (MLST), in general, did not lead to phylogenetic closeness by the GGI, which is perfectly logical, given that the GGI is a mobile element.

The results of our previous work on a GGI analysis of 150 isolates, which revealed a link between the susceptibility of isolates to ciprofloxacin, penicillin, and tetracycline and the presence of lesions in the GGI genes necessary for DNA secretion [20], were confirmed in this work on a sample of 9776 isolates. The influence of the presence of a functional GGI on the proportion of ciprofloxacin-, cefixime-, penicillin-, and tetracycline-resistant isolates in the population was established. The proportion of azithromycin-resistant isolates did not change when comparing isolates with a functional and a non-functional GGI.

The lack of statistical differences in the situation with azithromycin resistance and GGI functionality requires further investigation. Several hypotheses could be proposed as conceptual explanations. The first hypothesis is that different regions of the genome have an unequal capacity for horizontal transport. However, there are three regions with a low DUS density on the chromosome on the DUS distribution map [37], and none of them contain azithromycin resistance determinants. Moreover, *N. gonorrhoeae* strains with the meningococcal *mtrR* sequence are known [38], and the proportion of azithromycin-resistant isolates among samples with the meningitidis-like promoter is quite significant [39,40].

The second hypothesis is that the formation of a meaningful association between the presence of a functional GGI and antimicrobial resistance requires a time period sufficient for horizontal transmission and subsequent spread through the contact network via clonal reproduction. This assumption is supported by the fact that the use of azithromycin for therapy of a gonococcal infection was introduced later than cefixime and other antibiotics. Cefixime started to be used as a single-dose drug for the treatment of gonorrhea in England in 2005–2010 [41] and in the United States in the 2000s [42,43]. It is important to note that, during the same years, the CDC did not recommend the use of azithromycin as a gonorrhea treatment because of concerns about the development of resistance [43]. The widespread use of azithromycin as a component of dual therapy began in the United States in 2010, when the CDC recommended a single intramuscular dose of ceftriaxone (250 mg) and a single oral dose of azithromycin (1 g) [44]. The same regimen has been adopted in the UK and across Europe since 2012 [45,46].

The third hypothesis is that the resistance of *N. gonorrhoeae* to antimicrobials is not directly related to the presence/absence of a gonococcal island and may be associated with other mechanisms, for example, with the transduction of genetic resistance determinants by bacteriophages. Here, it should be noted that *N. gonorrhoeae* lacks the CRISPR-Cas system [47], which protects bacteria against the action of phages that can potentially facilitate viral DNA transfer.

Therefore, the type IV secretion system is significantly associated with the causative agent of gonorrhea exhibiting reduced susceptibility to the most of antimicrobial drugs. The presence of GGI may not only confer selective advantages to the bacterium in colonizing various niches [10] but may also contribute to the spread of antimicrobial resistance in the global gonococcal population. This probably applies not only to the resistance of *N. gonorrhoeae* to penicillin, tetracycline, ciprofloxacin, and cefixime, which we investigated in our work, but also to other drugs to be used in the future. The model and results presented here provide the basis for a sophisticated pathogen genotyping platform that combines standard typing approaches such as MLST, NG-MAST, and cgMLST with information on T4SS functionality and association with resistance to multiple antimicrobials.

## 5. Conclusions

Based on a sample of 9776 *N. gonorrhoeae* isolates from 63 countries, a model of GGI genetic diversity was proposed involving the division of the GGI of the global gonococcal population into 51 clusters and 3 superclusters based on the *traG* gene allele type and *atlA* and *ych* gene replacements for the *eppA* and *ych1* genes. The division reflects differences in the functionality of a type IV secretion system among isolates.

The NG-MAST typing system for 91% of the analyzed genomes allows one to make a conclusion about the presence of a GGI and of the GGI cluster and thus about the structure of the gonococcal island and its ability to secrete DNA. The distribution of GGI superclusters is globally not related to the phylogenetics of NG-MAST, indicating multiple horizontal transfers of the GGI.

A statistically significant difference in the proportion of *N. gonorrhoeae* isolates resistant to ciprofloxacin, cefixime, tetracycline, and penicillin was found when comparing populations with a functional and a non-functional GGI, which shows the importance of determining not only the presence of a GGI but also its ability to secrete DNA for predicting antimicrobial resistance. The proportion of isolates resistant to azithromycin is similar for isolates with a functional and a non-functional GGI.

## Data Availability

Not applicable. All data are in this manuscript and in the supplementary tables.

## Supplementary Materials

The following supporting information can be downloaded at: https://www.mdpi.com/article/10.3390/microorganisms11061547/s1, Table S1: Characteristics of GGI in 14,763 *N. gonorrhoeae* genomes, AMR, and typing data; Table S2: Presence of GGI genes in clusters; Table S3: Population characteristics of GGI genes; Table S4: (A) Distribution of NG-MAST types in GGI clusters, (B) Distribution of MLST types in GGI clusters; Table S5: NG-MAST and MLST heterogeneity in clusters; Table S6: Antimicrobial resistance among isolates with different GGI status; Figure S1: Distribution of downloaded *N. gonorrhoeae* genomes by country; Figure S2: Alignment of TraG proteins that are present in three GGI superclusters; Figure S3: NG-MAST heterogeneity in GGI clusters; Figure S4: MLST heterogeneity in GGI clusters.
