# Supplementary material for: Gonococcal Genetic Island in the Global Neisseria gonorrhoeae Population: A Model of Genetic Diversity and Association with Resistance to Antimicrobials
Source: Microorganisms. 2023 Jun 10;11(6):1547. doi: 10.3390/microorganisms11061547 (PMC10301925; doi:10.3390/microorganisms11061547)
Supplement: Supplementary file 1 [file microorganisms-11-01547-s001.zip › Figure S2. Alignment of TraG proteins that are present in 3 GGI superclusters.pdf]

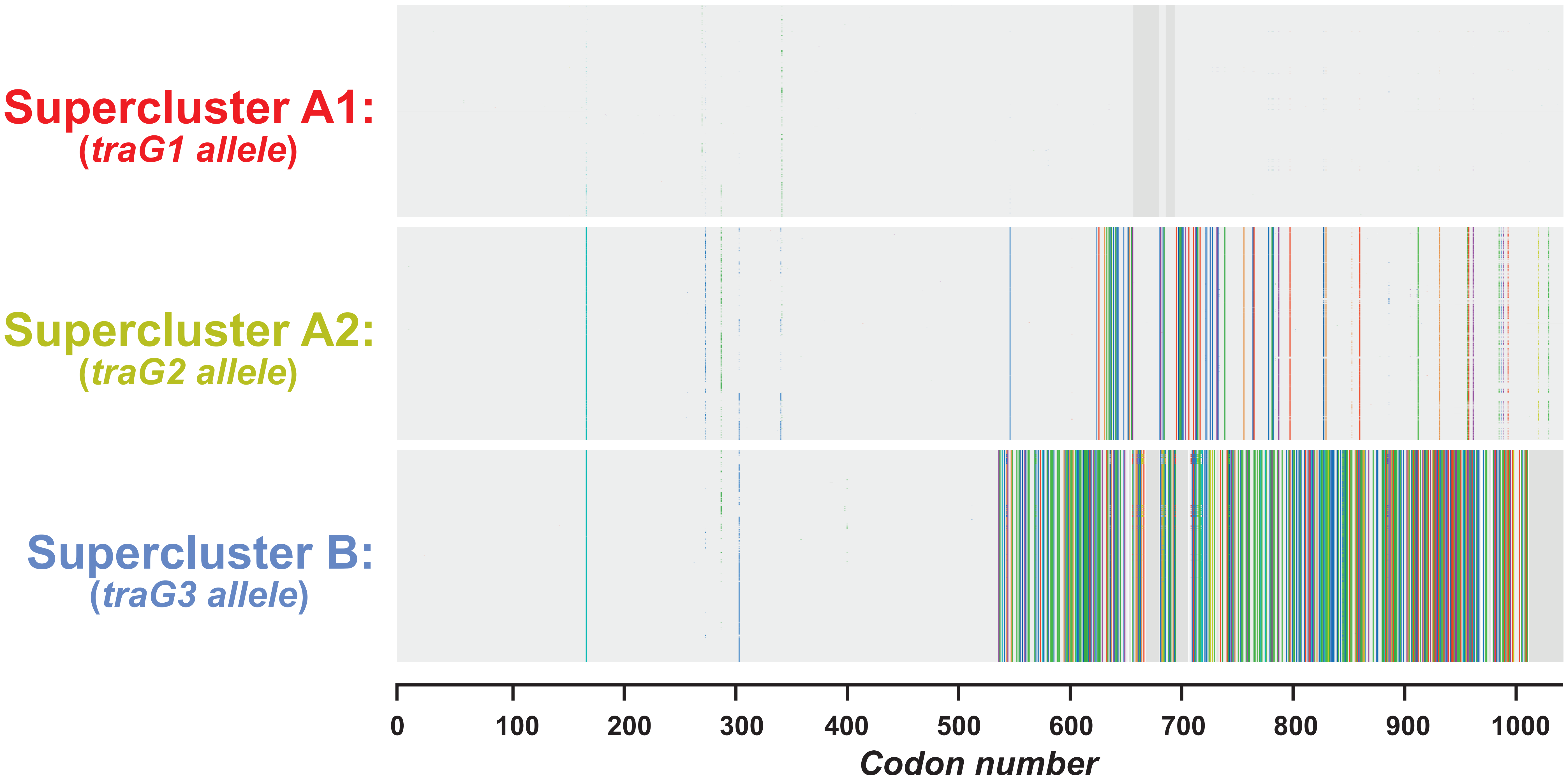

Figure S2. Alignment of TraG proteins that are present in three GGI superclusters. The light gray color indicates the absence of differences from the MS11 reference genome. Different bright colors indicate amino acid substitutions. Deletions/insertions are marked in dark gray.
