# Supplementary material for: Gonococcal Genetic Island in the Global Neisseria gonorrhoeae Population: A Model of Genetic Diversity and Association with Resistance to Antimicrobials
Source: Microorganisms. 2023 Jun 10;11(6):1547. doi: 10.3390/microorganisms11061547 (PMC10301925; doi:10.3390/microorganisms11061547)
Supplement: Supplementary file 1 [file microorganisms-11-01547-s001.zip › Figure S3. NG-MAST heterogeneity in GGI clusters.pdf]

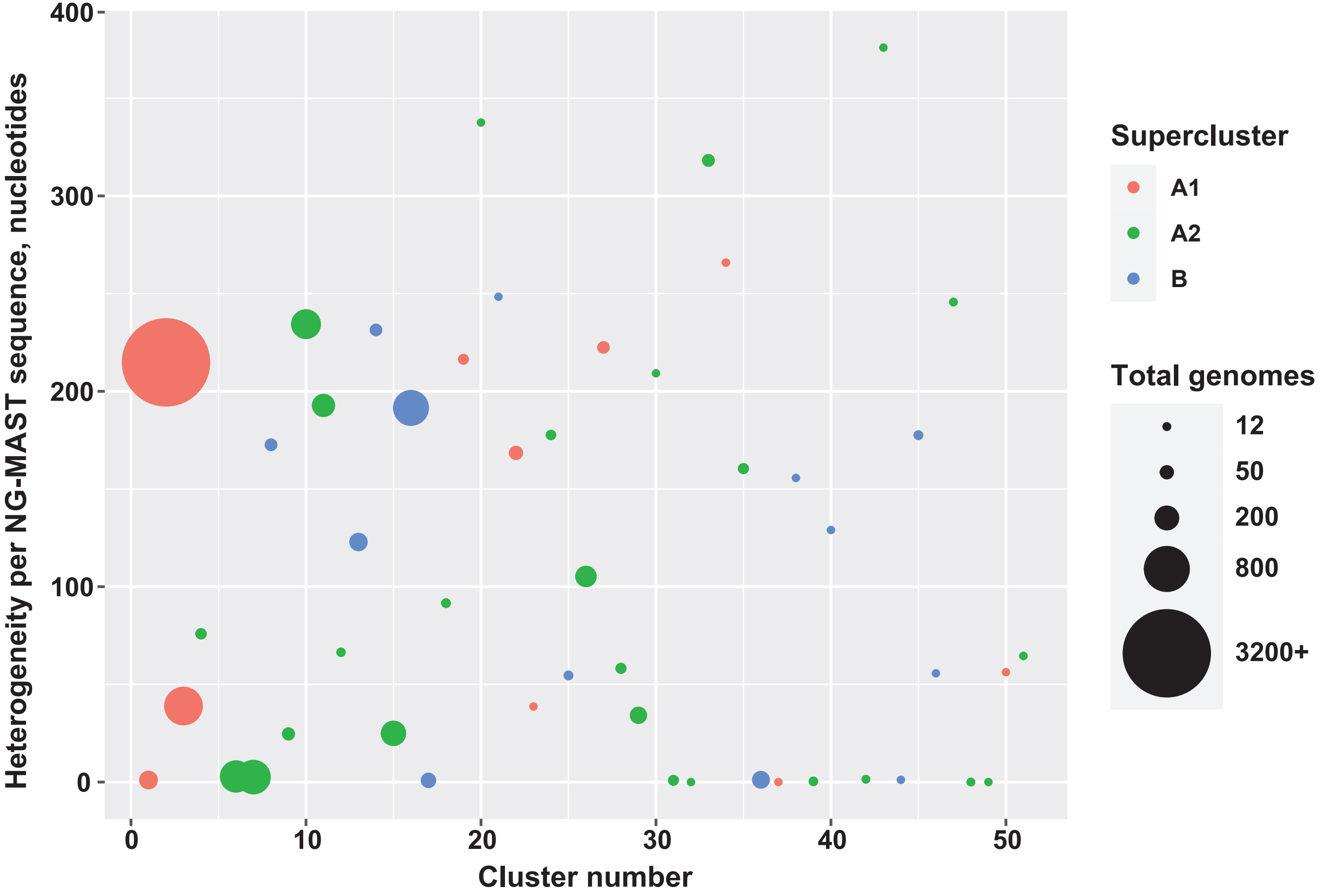

Figure S3. NG-MAST heterogeneity in GGI clusters. Red color - the cluster belongs to supercluster A1, green - to supercluster A2, blue - to supercluster B. The size of the circles shows the number of genomes in a cluster.
