# Supplementary figures and images for: Gonococcal Genetic Island in the Global Neisseria gonorrhoeae Population: A Model of Genetic Diversity and Association with Resistance to Antimicrobials
Source: Microorganisms. 2023 Jun 10;11(6):1547. doi: 10.3390/microorganisms11061547 (PMC10301925; doi:10.3390/microorganisms11061547)

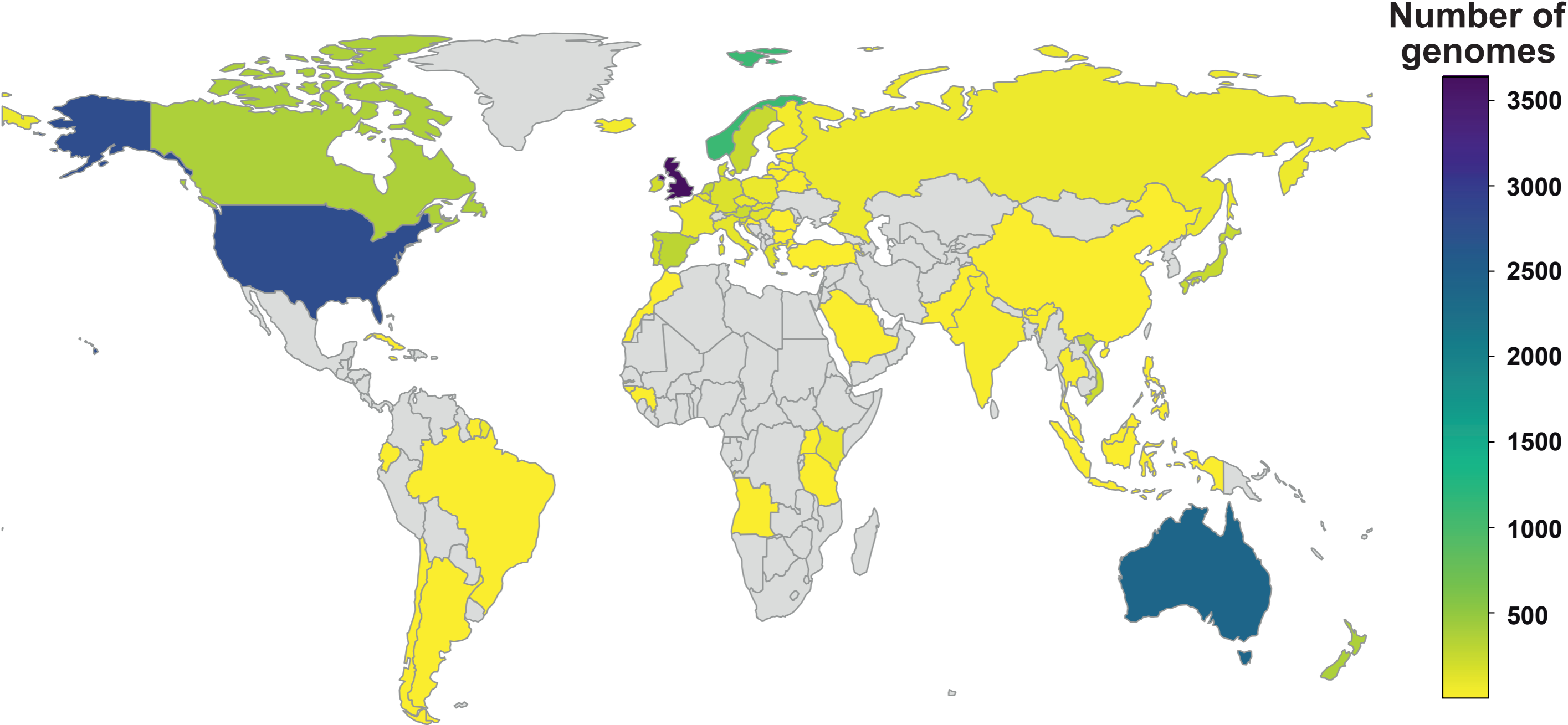

Figure S1. Distribution of downloaded *N. gonorrhoeae* genomes by country.

Supplement: Supplementary file 1 [file microorganisms-11-01547-s001.zip › Figure S1. Distribution of downloaded N. gonorrhoeae genomes by country.pdf]
